# Irrational Beliefs, Dietary Habits and 10-Year Incidence of Type 2 Diabetes; the ATTICA Epidemiological Study (2002-2012)

**DOI:** 10.1900/RDS.2021.17.38

**Published:** 2021-08-01

**Authors:** Christina Vassou, Mary Yannakoulia, Ekavi N. Georgousopoulou, Christina Chrysohoou, Christos Pitsavos, Mark Cropley, Demosthenes B. Panagiotakos

**Affiliations:** 1 Department of Nutrition and Dietetics, School of Health Sciences and Education, Harokopio University, Athens, Greece,; 2 DSchool of Medicine, Sydney, University of Notre Dame, Sydney, Australia,; 3 First Cardiology Clinic, School of Medicine, University of Athens, Greece,; 4 School of Psychology, University of Surrey, Guildford, UK

**Keywords:** irrational beliefs, type 2 diabetes, incidence, mediterranean diet, unhealthy lifestyle, depression, anxiety, mental health, literacy

## Abstract

**OBJECTIVE:**

The aim of this study was to evaluate the dietary habits and irrational beliefs of apparently healthy individuals in relation to their 10-year diabetes incidence.

**METHODS:**

The ATTICA study (2002-2012) is a prospective population-based cohort study, in which 853 participants (453 men (aged 45 ± 13 years) and 400 women (aged 44 ± 18 years)) without a history of cardiovascular disease (CVD) underwent psychological evaluations. Among other things, participants completed the Irrational Beliefs Inventory (IBI, range 0-88), a brief, self-reported measure consistent with the Ellis model of psychological disturbance. Demographic characteristics, detailed medical history, and dietary and other lifestyle habits were evaluated as well. Diagnosis of diabetes at follow-up examination was based on the criteria of the American Diabetes Association.

**RESULTS:**

Mean IBI score was 53 ± 10 in men and 51 ± 11 in women (p = 0.68). IBI was positively associated with the 10-year type 2 diabetes incidence (hazard ratio: 1.14; 95% CI: 1.04-1.25) in both men and women, and even more distinctly associated with participants with the following characteristics: lower education status, married, overweight, smokers, anxiety and depressive symptomatology, and unhealthy dietary habits. Especially, participants with increased irrational beliefs and low adherence to the Mediterranean diet were 37% more likely to develop type 2 diabetes than those with the reverse status (hazard ratio: 3.70; 95% CI: 2.32-5.88).

**CONCLUSIONS:**

These data support the need for lifestyle changes towards healthier nutrition which can be achieved by educating people so that they are equipped to recognize false and unhelpful thoughts and thus to prevent negative psychological and clinical outcomes such as mental health disorders and type 2 diabetes.

## 1 Introduction

Despite ongoing progress in treatment and management, diabetes remains a progressively growing disease [1]. The number of diabetic patients worldwide has more than doubled since 1980 and is predicted to reach 700 million adults by 2045 [2]. The global prevalence of diabetes among adults rose from 4.7% in 2000 to 8.5% in 2014 [1, 3]. These findings are critical since diabetes is a well-established cardiovascular risk factor [1]. The exponential increase of diabetes prevalence is also accompanied by a serious financial burden [1]. According to the International Diabetes Federation (IDF), the healthcare expenditure on diabetes in Europe five years ago was 2,198 euros per person per year [4].

At present, there is no curative treatment, making primary prevention the cornerstone of the global response to the disease [1]. Although genetic, biological, and environmental factors (in particular lifestyle interventions, including medical nutrition therapy, limited alcohol intake, regular physical activity, etc.) are decisive in the onset and development of diabetes, cognitive and psychosocial management are also crucial in both preventing the disease and improving the daily life of diabetic patients [1, 5].

In cognitive-behavioral therapy, irrational beliefs are considered as the primary cause of psychopathological conditions, namely anxiety, depression, eating problems (including overeating, unhealthy diet, obesity, etc.), and alcohol overconsumption [6]. Specifically, irrational beliefs derive from a core process of absolutist thinking and pessimism (e.g., “I am a boring person, nobody wants to talk to me”) which inevitably contribute to the development of psychopathologic conditions, including stress and extreme sadness. Irrational coping strategies may lead to irrational behavioral responses, such as misuse of alcohol and eating problems (e.g., “I will drink a few glasses of whisky to make me feel less shy and more comfortable with others”), which may contribute to the development of type 2 diabetes, cardiovascular disease, and mortality [7-10]. Anxiety and depression have also been associated with certain eating habits, such as significantly less fruit, vegetable, and fish intake, and increased sugar consumption [10]. Depression has also been associated with low consumption of folic acid, omega-3 fatty acids, folate, cobalamin, zinc, magnesium, B-group vitamins, and culinary fat, whereas anxiety has been associated with lower consumption of docosahexaenoic acid (DHA), one of the ω-3 long-chain polyunsaturated fatty acids (LC-PUFAs) [11, 12]. High amounts of fat and soft drinks and high consumption of red meat, sweets, and fried foods may result in the deterioration of glucose tolerance and insulin sensitivity raising the risk of diabetes, whereas whole grains, nuts, legumes, fruits, vegetables, micronutrients, and low alcohol intake act protectively [13-15].

Although the biological association of anxiety and depression in type 2 diabetes has been proposed by a significant number of studies, the mechanisms underlying this association are still unclear [16]. Besides, the literature on anxiety, depression, and nutrition is relatively limited. To date, no studies have examined the association between irrational beliefs and dietary habits, and how their synergistic role may contribute to the incidence of type 2 diabetes. Therefore, the present study aimed to evaluate the association between irrational beliefs and dietary habits, as well as the combined role of irrational beliefs and the Mediterranean diet in the 10-year incidence of type 2 diabetes.

## 2 Methods

### 2.1 Study design

The ATTICA study is a population-based, prospective survey (2002-2012) which was conducted in the province of Attica, in Greece. Briefly, 3,042 adults (18-89 years old, 49% men, 73% participation rate) without any clinical evidence of cardiovascular diseases, other atherosclerotic impairments, or chronic viral infections agreed to provide blood samples for biochemical and genetic analyses in addition to the requested sociodemographic, lifestyle, and medical information, including a psychological evaluation.

#### Setting

The study was conducted in the greater metropolitan Athens area (including 78% urban and 22% rural regions) during 2001-2002. The participant’s examination was performed using face-to-face interviews, in the individual’s home or workplace, by trained personnel (cardiologists, general practitioners, dieticians and nurses, as well as psychiatrists/psychologists).

### 2.2 Patient sample

Of the initially enrolled 3,042 participants, a subsample of 853 participants (453 men (45 ± 13 years) and 400 women (44 ± 18 years)) agreed to participate in the psychological evaluation. This subsample was representative of the total study sample since there were no differences in sex and age distribution between the studied sample and the overall study population (all p-values >0.4).

### 2.3 Bioethics

The study was approved by the Institutional Ethics (#017/1.5.2001) Committee. All participants were informed about the aims and procedures and agreed to participate by providing written consent.

### 2.4 Sociodemographic and lifestyle measurements at baseline

Sociodemographic and lifestyle characteristics assessed included age, sex, educational level, mean annual income during the past three years, dietary habits, the level of adherence to the Mediterranean diet, physical activity status, and smoking habits. Socioeconomic status (SES) was classified into three groups (tertiles; low, medium, and high) according to the SES categorization already used, taking into account education level and mean annual income in the preceding three years [17]. Current smokers were classified as individuals who had smoked at least one cigarette per day during the previous year, former smokers were defined as those who had stopped smoking more than 1 year ago, and the rest were classified as “never-smokers”.

Dietary habits were recorded using a validated semiquantitative food-frequency questionnaire (FFQ), the EPIC-Greek questionnaire, which was kindly provided by the Unit of Nutrition of Athens University Medical School, and asked participants to recall and report the average weekly or daily intake of food items during the past year [18]. The questionnaire recorded the usual dietary intake of 156 foods and beverages commonly consumed in Greece, with seven non-overlapping response categories. Photographs assisted the responders to define the portion sizes in several foods that were included in the questionnaire. In particular, consumption of non-refined cereals and products (like whole-grain bread, pasta, rice, etc.), vegetables, legumes, fruit, dairy products (like cheese, yogurt, milk), nuts, potatoes, eggs, sweets, fish, poultry, red meat and meat products, use of olive oil in cooking, as well as coffee, tea, and alcohol drinking were measured as an average per week during the past year. The frequency of consumption was quantified in terms of the number of times per month a food item was consumed in small, medium, or large portion sizes.

Alcohol consumption was recorded as daily ethanol intake of 100 ml wineglasses adjusted for 12% ethanol concentration. Moreover, a diet score, called MedDietScore, was calculated based on the reported dietary habits to assess adherence to the Mediterranean diet for each participant [18]. In particular, for the consumption of items presumed to be “close” to this pattern (i.e., those consumed daily or more frequently than 4 servings per week) we assigned the score 0 when someone reported no consumption, score 1 for 1 to 4 times per month, score 2 for 5 to 8 times per month, score 3 for 9 to 12 times per month, score 4 for 13 to 18 times per month and score 5 for more than 18 times per month. For the consumption of food items presumed to be outside of this diet (like meat and meat products) we assigned the scores on a reverse scale (i.e., 0 when a participant reported almost daily consumption and 5 for rare or no consumption). Specifically for alcohol, we assigned score 5 for the consumption of fewer than 3 wineglasses per day, score 0 for the consumption of more than 7 wineglasses per day and scores 1 to 4 for the consumption of 3, 4-5, 6, and 7 wineglasses per day. Higher values (range 0-55) of this special diet score mentioned above indicate good adherence to the Mediterranean diet.

A short form of the International Physical Activity Questionnaire (IPAQ) was used to assess physical activity. The IPAQ was used as an index of weekly energy expenditure using frequency (times per week), duration (in minutes per time), and intensity of sports or other habits related to physical activity (in expended calories per time) [19]. Participants who did not report any physical activities were defined as sedentary and the rest as physically active.

### 2.5 Clinical and biochemical evaluation at baseline

Blood samples were collected from the antecubital vein at between 8 to 10 am in a sitting position after 12 hours of fasting and alcohol abstinence. Diagnosis of type 2 diabetes mellitus was based on the criteria of the American Diabetes Association [20]; i.e., participants who had fasting blood glucose >125 mg/dl during the examination or reported the use of antidiabetic medication were defined as having diabetes. Blood glucose levels (mg/dl) were measured with a Beckman Glucose Analyzer (Beckman Instruments, Fullerton, CA, USA). Serum insulin concentrations were assayed using radioimmunoassay (RIA100, Pharmacia Co., Erlangen, Germany). Moreover, obesity was defined as body mass index (BMI) greater than 29.9 kg/ m2 according to WHO criteria [21]. Waist and hip circumferences (in cm) were also measured; and waist-to-hip (WH) and waist-to-height (WHt) ratios were calculated. WH ratio was considered abnormal if >0.8 for women and >1 for men, whereas WHt ratio was considered abnormal if >0.5 for both sexes. Regarding other clinical characteristics, arterial blood pressure (3 recordings) was measured at the end of the physical examination with the subject in a sitting position after at least 30 minutes at rest. Participants whose average blood pressure levels were ≥140/90 mmHg and who were taking antihypertensive medication were classified as being hypertensive. Hypercholesterolemia was defined as total cholesterol levels >200 mg/dl or the use of lipid-lowering agents. The intra- and inter-assay coefficients of variation of cholesterol levels did not exceed 9%. The metabolic syndrome was defined according to ATP III criteria [22].

### 2.6 Psychological evaluation at baseline

Irrational beliefs were assessed at baseline using the Irrational Beliefs Inventory (IBI), a brief self-report measure based on the work of Ellis [23]. The inventory consists of 11 statements, each reflecting one irrational belief, including, worrying, rigidity, need for approval, problem avoidance, and emotional irresponsibility [24]. Each item is followed by a 9-point bipolar scale ranging from disagree to agree. The scales are added up to yield a total score ranging from 0 to 88 (the higher the score, the more irrational the beliefs).

The IBI was developed as an instrument to assess the association between endorsement of irrational beliefs and various aspects of maladaptive emotion and behavior that have been developed within Ellis’s theoretical and applied model that views irrational beliefs as maladaptive [23]. For this study, the IBI score was subdivided into 3 categories with the 1^st^ tertile corresponding to the lowest IBI scores (<48, i.e., less irrational beliefs), the 2^nd^ to the moderate IBI scores (48-56) and the 3^rd^ to the highest IBI scores (>56, i.e., frequent irrational beliefs).

Depressive symptomatology was assessed using the validated Greek translation of the Zung Self-Rating Depression Scale (ZDRS). The time window was the preceding 4-week period before administration. The ZDRS total score range is 20-80, with higher score values indicating more severe depression symptoms [25]. Based on the validated ZDRS cut-off score for the Greek population, we applied a cut-off score of 45 to dichotomize the study cohort into participants with and without clinically relevant depressive symptomatology [26].

Anxiety levels were assessed using the validated Greek translation of the State Anxiety subscale of the Spielberger State-Trait Anxiety Inventory (STAI) [27]. The total score of the 20-item STAI ranges from 20 to 80, with higher score values indicating more severe anxiety symptoms [25]. In the context of this study, the STAI score was used as a continuous variable, since cut-off scores for the adult Greek population require further validation [27].

### 2.7 10-year follow-up examination

The ATTICA study 10-year follow-up was performed during 2011-2012. Of the initially enrolled 3,042 participants at baseline, a 10-year follow-up was achieved in 2,583 participants (85% participation rate; of those lost to follow-up, n = 224 could not be traced due to missing contact information and n = 235 stopped participating). After excluding patients diagnosed with diabetes at baseline examination (n = 210), and those that did not attend the psychological examination (n = 1,528), the working sample consisted of n = 845 participants. Diagnosis of diabetes at follow-up examination was based on the American Diabetes Association criteria mentioned above [20].

No differences were found regarding the distribution of sex, years of education, family history of diabetes, physical activity status, metabolic syndrome, hypercholesterolemia status, BMI, abnormal waist-tohip and waist-to-height ratio, and energy intake (all p-values >0.05). Some significant differences in baseline characteristics were observed between participants for whom information about 10-year diabetes status was available and the rest; in particular, compared to those lost to follow-up, participants had a slightly higher prevalence of hypertension (30% vs. 26%, p = 0.036), smoking habits (58% vs. 54%, p = 0.028), and abnormal waist circumference (50% vs. 54%, p = 0.027). They also had higher fasting glucose levels (88 ± 12 vs. 80 ± 13 mg/dl, p = 0.005) and lower fasting insulin levels (12 ± 3.0 vs. 13 ± 3.4 μU/ml, p = 0.014).

### 2.8 Study size and statistical power analysis

Power analysis showed that the number of participants in the working dataset was adequate to evaluate two-sided differences between subgroups and that the investigated parameters were greater than 20%, achieving statistical power >0.80 at <0.05 probability level (p-value).

### 2.9 Statistical methods

Incidence (and the corresponding 95% confidence interval) of diabetes was calculated as the ratio of new cases to the number of people that participated, who were free of diabetes at baseline, and participated in the follow-up. Continuous variables are presented as mean values ± standard deviation, and categorical variables are presented as frequencies. Associations between categorical variables were tested using the chi-squared test. Comparisons of mean values of normally distributed variables between those who developed diabetes and the rest of the participants were performed using Student’s t-test, after controlling for equality of variances using Levene’s test.

For continuous variables that were not distributed normally (e.g., years of school) the Mann-Whitney non-parametric test was applied to evaluate differences in the distribution of the skewed variables. Continuous variables were tested for normality using P-P plots. Associations between normally distributed variables and IBI tertiles were evaluated through a one-way analysis of variance (ANOVA). For non-normally distributed variables, the Kruskal-Wallis test was used. Associations between categorical variables and IBI tertiles were assessed using the chi-squared test. Since the exact time of the event (i.e., development of diabetes) was unknown, the relative risks of developing the disease during the 10-year follow-up period were estimated using the odds ratios (OR) and their corresponding 95% confidence intervals (CIs) through stepwise multiple logistic regression analysis. Interactions between sex and other covariates were tested in all steps, and when significant, they remained in the final model.

The Hosmer-Lemeshow test was applied to evaluate models’ goodness-of-fit. The -2 log-likelihood ratio of the initial vs. the final model was also calculated to evaluate the models’ performance (the lower the better). All known confounders were included in the models after testing for collinearity. The Statistical Package for Social Sciences (SPSS) version 25 (SPSS Inc., Chicago, IL, U.S.A.) software was used for all statistical calculations.

## 3 Results

### 3.1 Baseline associations of participants’ characteristics with irrational beliefs

Mean IBI score was 53 ± 10 for men and 51 ± 11 for women (p = 0.68). Moreover, 33% of the participants had an IBI score of <48/80 (1^st^ tertile), whereas 67% had a score of >56/80 (3^rd^ tertile), i.e., frequent irrational beliefs. As can be seen in Table 1, people in the highest tertile (i.e., 3^rd^) of IBI were older, more likely to be smokers, married, less educated (as they reported fewer school-years), and had lower annual income (p < 0.001). Also, people with a high level of irrational beliefs (i.e., 3^rd^ tertile) had higher BMI, higher prevalence of obesity, and were more likely to have depression or anxiety symptoms (p < 0.001). No association between irrational beliefs status and sex, sedentary lifestyle, certain clinical characteristics such as history of hypertension and hypercholesterolemia, or family history of diabetes was observed (all p-values >0.10) (Table 1).

**Table 1.** Baseline sociodemographic, lifestyle, and clinical characteristics of the ATTICA study participants according to IBI tertiles (n = 845)

Characteristic	Overall sample		IBI tertiles		p
		1st (low)	2nd (moderate)	3rd (high)	
Age (yr.)	39 ± 11	40 ± 10	41 ± 10	47 ± 8	0.001
Male sex (%)	52	47	52	55	0.17
Years of school	13±3	14±2.5	13±3	11±3	<0.001
Financial status (% low)	45	42	44	49	<0.001
Marital status (% married)	66	62	68	70	<0.001
Current smoking (%)	41	36	47	47	<0.001
Cigarette packages (per yr.)	380	399	379	357	0.272
Sedentary life (%)	54	51	59	52	0.12
Obesity (%)	48	47	54	55	<0.001
Body mass index (kg/m2)	25±4	25±4	26±4	27±4	<0.001
Family history of diabetes (%)	25	22	30	27	0.05
Hypertension (%)	24	23	26	23	0.28
Hypercholesterolemia (%)	31	27	33	34	0.004
ZUNG (0-80) a	35±7	33±6	36±7	38±6	<0.001
STAI (0-80) b	40±12	35±10	41±11	45±12	<0.001

**Legend:** Data are presented as mean ± standard deviation (SD), i.e., mean (SD). p-values were obtained using one-way analysis of variance (ANOVA) for independent samples for the normally distributed variables, the Kruskal-Wallis Test for the remaining quantitative variables and the chi-squared test for categorical variables. ^a^ Zung Self-Rating Depression Scale. ^b^ State-Trait Anxiety Inventory.

### 3.2 Food group consumption and nutrient intake according to IBI status at baseline

In Table 2, the consumption of specific food groups is presented by the level of IBI tertile. Data analysis revealed that, overall, participants in the lowest IBI tertile (fewer irrational beliefs) had greater adherence to the Mediterranean diet than those in the highest tertile (p < 0.001). This finding was further supported by the fact that participants in the lowest irrational beliefs group used olive oil in daily cooking almost exclusively, consumed more vegetables and potatoes, less red meat and its products, and consumed fewer soft drinks and coffee than those in the highest tertile who seemed to practice unhealthier dietary habits (all p values <0.05).

**Table 2.** Food group consumption and nutrient intake of the ATTICA study participants according to IBI tertiles (n=845)

		IBI scale tertiles		p
	1st (low)	2nd (moderate)	3rd (high)	
MedDietScore: 0-55 (n)	27.5 ± 8	27.0 ± 9	25.6 ± 7	<0.001
Vegetables (servings/wk)	38 ± 11	36 ± 10	33 ± 11	0.05
Legumes (servings/wk)	5 ± 2	5 ± 2	5 ± 2	0.98
Non-refined cereals and products (servings/wk)	53 ± 17	54 ± 16	56 ± 18	0.94
Potatoes (servings/wk)	10 ± 6	11 ± 5	12 ± 7	<0.001
Fruits (servings/wk)	27 ± 13	25 ± 12	27 ± 12	0.12
Dry fruits (servings/wk)	7 ± 7	6 ± 4.8	7 ± 6.5	0.40
Red meat (servings/wk)	4.4 ± 2.2	5.5 ± 2.8	6.4 ± 2.5	0.007
Poultry (servings/wk)	1.2 ± 0.7	1.2 ± 0.9	1.3 ± 0.8	0.61
Fish and fisheries (servings/wk)	2 ± 0.8	2 ± 1	2 ± 2	0.19
Eggs (servings/wk)	1 ± 0.7	1 ± 0.7	1 ± 1	0.82
Nuts (servings/wk)	1.7 ± 1.8	1.4 ± 1.2	2 ± 1.8	0.36
Sweets (servings/wk)	5 ± 2	5 ± 4	5 ± 2	0.63
**Fats and oils**				
Olive oil (daily use, %)	99	100	95	<0.001
Seed oil (daily use, %)	0.7	0.8	0.8	0.54
Butter (daily use, %)	93	93	99	0.87
Margarine (daily use, %)	7	11	12	0.44
**Soft drinks**				
Soft drinks (glasses/day)	2.5 ± 2.5	2.5 ± 2.7	3.7 ± 2.8	0.05
Cola drinks (glasses/day)	2.1 ± 1.7	1.6 ± 1.8	2.4 ± 2	0.06
**Other drinks**				
Coffee (cups/day)	1.3 ± 0.4	1.4 ± 0.5	1.4 ± 0.5	0.05
Tea (cups/day)	0.4 ± 0.4	0.4 ± 0.3	0.5 ± 0.7	0.65
Alcohol (g/day)	8 ± 12	11 ± 19	9 ± 15	0.25
Beer (glasses/day)	2 ± 0.8	2 ± 0.8	2 ± 0.8	0.03
Wine (glasses/day)	2 ± 0.8	2 ± 0.8	2 ± 0.8	<0.001

### 3.3 Irrational beliefs, baseline characteristics, and 10-year incidence of type 2 diabetes

During 2002-2012, the crude 10-year incidence of diabetes in the working sample was n = 191 cases (12.9%, 95%CI: 10.4-15.4) or 129 per 1,000 participants free of diabetes at baseline. Of the latter, n = 97 (13.4%, 95%CI: 10.8-16) were men and n = 94 (12.4%, 95%CI: 10.1-14.7) were women. Stratified analysis by age group revealed that the incidence of diabetes was the lowest, i.e., 1.5%, for people under 35 years old, but increased to 29.2% for participants over 74 years old. The men-to-women incidence was approximately 1-to-1 in almost all age groups.

Participants who developed diabetes during the 10-year follow-up had an 11.8% higher IBI score at baseline examination than those who did not develop diabetes (i.e., 57/80 vs. 51/80) (Table 3). Furthermore, the 10-year incidence of diabetes was 5% among those in the lowest IBI tertile compared to 7.8% among those in the middle and 7.5% among those in the highest tertile (p < 0.001). Crude, unadjusted data analysis revealed that participants in the 3^rd^ tertile of IBI scores had a 14% higher risk of developing diabetes during the 10-year follow-up than those in the 1^st^ tertile (OR: 1.14, 95%CI: 1.04-1.25). A progressive, but insignificant increase was also observed between participants in the 2^nd^ tertile compared to those in the 1^st^ tertile (OR: 1.08, 95% CI: 0.94-1.28). Further analysis revealed that people who developed diabetes were older at baseline examination, had lower education, financial and SES status, higher fasting glucose levels, were more likely to be predisposed to diabetes because of family status, and had a history of hypertension and hypercholesterolemia.

**Table 3.** Baseline characteristics of the ATTICA study participants according to the 10-year diabetes incidence (n = 845)

Baseline characteristic	Status at 10-year follow-up		p
	Without diabetes	With diabetes	
Age (yr.)	44 ± 13	53 ± 11	<0.001
Male sex (%)	49	51	0.57
SES (low, %)	6	18.5	<0.001
**Lifestyle**			
Current smokers (%)	54	52	0.62
Physically active (%)	43	38	0.25
**Medical history**			
Fasting glucose (mg/dl)	88 ± 12	95 ± 14	<0.001
Hypertension (%)	27	46	<0.001
Hypercholesterolemia (%)	37	56	<0.001
Family history of diabetes (%)	20	36	<0.001
Body mass index (kg/m2)	26 ± 4.0	29 ± 5.0	<0.001
Waist circumference (cm)	88 ± 14	98 ± 16	<0.001
Abnormal WHR ratio * (n)	34	59	<0.001
IBI: 22-88 (n)	51 ± 11	57 ± 9	0.001
ZDRS: 0-80 (n)	35 ± 7.0	38 ± 10	0.68
STAI: 0-80 (n)	40 ± 12	42 ± 14	<0.001
**Dietary habits**			
MedDietScore: 0-55 (n)	27 ± 8.0	26 ± 10	0.01
Vegetables (servings/wk)	35 ± 13	38 ± 17	0.41
Legumes (servings/wk)	5 ± 2.3	5 ± 2.4	0.94
Cereals (servings/wk)	53 ± 18	56 ± 20	0.40
Red meat (servings/wk)	4.5 ± 2.4	3.6 ± 1.2	0.22
Poultry (servings/wk)	1.30 ± 0.8	1.23 ± 0.7	0.73
Fish (servings/wk)	2 ± 0.9	2.5 ± 1	0.03

**Legend:** * >0.8 for women and 1 for men.

Regarding anthropometric characteristics, participants who developed diabetes during the 10-year follow-up had higher BMI, higher waist circumference values, and abnormal WH and WHt ratios, and were more likely to have metabolic syndrome at baseline examination. Moreover, participants who developed diabetes during the follow-up reported lower adherence to the Mediterranean diet at baseline evaluation (all p-values <0.05), but no other associations with food groups or drinks consumed and 10-year diabetes incidence were observed (Table 3).

### 3.4 Multistage analysis of irrational beliefs and 10-year incidence of type 2 diabetes

As already mentioned, crude analysis revealed a positive association between IBI score and the risk of developing diabetes during the 10-year follow-up. However, residual confounding may exist, especially when several associations between IBI score and age, psychological factors, and dietary habits of the participants have already been observed. Thus, after adjustment for sex and age, IBI score was still significantly associated with the 10-year incidence of type 2 diabetes; specifically, a 1-unit increase in IBI score led to an 11% increase in type 2 diabetes risk (Table 4, Model 1). The latter association remained significant even after adjusting for SES and smoking habits of the participants (Models 2 and 3, Table 4).

**Table 4.** Results from nested, multi-adjusted models that evaluated the association between Irrational Beliefs Index (IBI) score and 10-year incidence of diabetes

IBI score	Model 1	Model 2	Model 3	Model 4	Model 5	Model 6
*2nd vs. 1st tertile*	1.06 (0.99, 1.15)	1.43 (1.11, 1.94)	1.32 (1.02, 1.80)	1.29 (0.99, 1.61)	1.24 (0.91,1.55)	1.22 (0.78, 1.94)
*3rd vs. 1st tertile*	1.11 (1.04, 1.19)	1.44 (1.12, 1.98)	1.33 (1.07, 1.74)	1.31 (0.98, 1.76)	1.30 (0.95,1.78)	1.30 (0.94, 1.81)
SES (high vs. low tertile)		0.36 (0.13, 0.98)	0.38 (0.14, 1.11)	0.37 (0.13, 1.06)	0.49 (0.17,1.43)	0.46 (0.08, 2.52)
Current smoking (Y/N)		1.11 (0.88, 1.42)	1.12 (0.92, 1.38)	1.17 (0.99, 1.44)	1.18 (1.01, 1.54)	1.21 (1.02, 1.58)
Body mass index (per 1 kg/m2)			1.14 (1.08, 1.20)	1.14 (1.09, 1.21)	1.12 (1.04,1.20)	1.12 (1.05, 1.20)
Physical inactivity (Y/N)			1.10 (0.70, 1.74)	1.12 (0.71, 1.76)	1.16 (0.71,1.95)	1.21 (0.71, 1.99)
MedDietScore (per 1 unit)				0.97 (0.94,0.99)	0.99 (0.95,1.02)	0.98 (0.94, 1.00)
Hypertension (Y/N)					1.33 (0.84, 2.17)	1.35 (0.82, 2.22)
Hypercholesterolemia (Y/N)					1.96 (1.31, 2.69)	1.51 (1.21, 1.82)
Family history of diabetes (Y/N)					2.57 (1.76, 3.77)	2.59 (1.71, 3.79)
ZDRS (per 1 unit)						1.05 (0.99, 1.11)
STAI (per 1 unit)						1.01 (0.97, 1.05)

**Legend:** Data are presented as hazard ratios and 95% confidence intervals All models are age- and sex-adjusted. * p-values <0.05. ZDRS (Zung Self-Rating Depression Scale) and STAI (State-Trait Anxiety Inventory) were entered separately in Model 6.

After including several clinical variables in the model, IBI was no longer associated with a 10-year risk of diabetes in all scenarios, underlying the confounding role of these factors in the relationship between irrational beliefs and risk of type 2 diabetes mellitus (see Models 4-6, Table 4). Moreover, no significant interactions between IBI score and physical activity, smoking, obesity, history of hypertension, and hypercholesterolemia on 10-year diabetes outcome were observed (all p-values >0.10).

However, a highly significant interaction was observed between MedDietScore and IBI on 10-year diabetes risk (p < 0.001). Thus, the analysis was stratified by the level of adherence to the Mediterranean diet and IBI score. As can be seen in Table 5, in the crude model, participants with high IBI and high adherence to the Mediterranean diet had a 2.5-times higher likelihood of developing diabetes during the 10-year follow-up than participants who highly adhered to the Mediterranean diet, but had a low level of irrational beliefs, underlying the harmful effect of irrational beliefs on diabetes development among participants with healthy dietary habits. Also, participants with a low level of irrational beliefs and also low adherence to the Mediterranean diet had a 1.69-times higher likelihood of developing diabetes during the 10-year follow-up than participants who highly adhered to the Mediterranean diet, but had a low level of irrational beliefs. Similarly, participants with high IBI, but low adherence to the Mediterranean diet had a 3.44-times higher likelihood of developing diabetes than participants who highly adhered to the Mediterranean diet, but had a low level of irrational beliefs, underlying the combined harmful, additive effects of both irrational beliefs and practicing unhealthy dietary pattern.

**Table 5.** Nested logistic regression analysis models to evaluate the association of combined irrational beliefs and Mediterranean diet adherence with 10-year incidence of type 2 diabetes

Combined IBI scale and Med- DietScore	Crude model	Age and sex- adjusted model	Multi-adjusted model		
			Model 1	Model 2	Model 3
Low IBI score & high MedDiet- Score	Reference	Reference	Reference	Reference	Reference
High IBI score & high MedDiet- Score	2.50 (1.61, 3.84)	2.00 (1.28, 3.12)	1.92 (1.21, 3.03)	2.56 (1.47, 4.54)	2.56 (1.47, 4.54)
Low IBI score & low MedDietScore	1.69 (1.12, 2.56)	1.78 (1.16, 2.77)	1.96 (1.26, 3.03)	2.17 (1.28, 3.70)	2.17 (1.28, 3.70)
High IBI score & low MedDiet- Score	3.44 (2.22, 5.26)	3.44 (2.17, 5.55)	3.70 (2.32, 5.88)	3.44 (1.88, 6.25)	3.46 (1.92, 6.25)

**Legend:** Data are presented as hazard ratios and 95% confidence intervals. ORs and their 95%CIs were obtained through logistic regression analysis. **Model 1** was adjusted for age, sex, educational status, physical activity, and current smoking. **Model 2** was additionally adjusted for baseline BMI, fasting blood glucose, and family history of diabetes. **Model 3** was additionally adjusted for the Zung Self-Rating Depression Scale.

Subsequently, the models were adjusted for age, sex, BMI, physical activity status, and smoking habits, and the results regarding IBI and 10-year diabetes incidence remained significant (Models 1 and 2, Table 5). When the models were further adjusted for baseline fasting blood glucose and family history of diabetes and additionally adjusted for depression or anxiety status (through the ZDRS, STAI) (Model 3, Table 5), it was revealed that participants with a high level of irrational beliefs and high adherence to the Mediterranean diet had a 2.56-times higher likelihood of developing diabetes than participants with a low level of irrational beliefs, but high adherence to Mediterranean diet. In accordance, participants with low IBI and low adherence to the Mediterranean diet had a 2.17-times higher likelihood of developing diabetes than participants who highly adhered to the Mediterranean diet, but had low levels of irrational beliefs. Finally, participants with high IBI and low adherence to Mediterranean diet had a 3.44 times higher likelihood of developing diabetes than participants who highly adhered to the Mediterranean diet and had low levels of IBI, underlying the combined harmful effect of negative thinking patterns and unhealthy dietary habits on the development of type 2 diabetes.

As people’s irrational beliefs and attitudes are usually considered as precursors of negative emotions, like depression and anxiety, the additive effect of these psychological disorders was further evaluated. As shown in Table 1, participants in the highest tertile on the IBI scale also had higher levels of depression (p < 0.001) and anxiety (p < 0.001) than those in the lowest tertile. When the analysis was focused on those in the upper 50 percentile of the depression score (i.e., ZDRS > 35) it was observed that participants in the 3^rd^ IBI tertile were 1.25-times more likely to develop diabetes than those in the lowest IBI tertile (95% CI: 0.98-2.27) after adjusting for the same factors as in Model 5 (Table 4). Furthermore, when the analysis was focused on participants in the lowest 50 percentile (i.e., ZDRS ≤ 35), no association between IBI score and diabetes incidence was observed (OR: 1.12, 95% CI: 0.43-2.91). Analysis then focused on participants in the upper 50 percentile of the anxiety score (i.e., STAI > 40). It was observed that individuals in the 3^rd^ IBI tertile had a 1.41-times higher likelihood of developing diabetes than those in the lowest IBI tertile (95% CI: 1.12-2.89), while no association between IBI score and diabetes incidence was observed (OR: 0.64, 95% CI: 0.22-1.83) when the analysis was focused on those in the lowest anxiety score 50 percentile (i.e., STAI ≤ 40).

## 4 Discussion

In this study, lifestyle and clinical profile of people with irrational beliefs and their relationship with the 10-year diabetes incidence were explored. It was revealed that people who reported more irrational beliefs had a lower SES, with less education and income, had more mental and physical health conditions (e.g., anxiety, depression, high BMI, and obesity), practiced unhealthier dietary habits, and experienced a higher 10-year risk of developing type 2 diabetes mellitus. Moreover, people reporting fewer irrational beliefs showed a greater adherence to the Mediterranean diet, consumed more vegetables and less red meat and its products, and drunk fewer soft drinks.

This is the first study to demonstrate that irrational beliefs contribute to the 10-year incidence of type 2 diabetes. Participants who strongly pursued irrational beliefs and had a low adherence to the Mediterranean diet were 37% more likely to develop diabetes than those with a low level of irrational beliefs status and high adherence to the Mediterranean diet. Finally, participants who eventually developed type 2 diabetes during the 10-year follow-up period had an 11.8% higher irrational beliefs score at baseline, higher BMI, abnormal WH and WHt ratios, lower adherence to the Mediterranean diet, history of hypertension, hypercholesterolemia, and metabolic syndrome than those who did not develop diabetes. Despite the limitations of this work inherent in its observational nature, it carries significant public health messages, since it suggests, for the first time in the literature, that irrational beliefs play a role in the development of type 2 diabetes in combination with unhealthy dietary patterns and non-adherence to the Mediterranean diet.

Type 2 diabetes is a complex metabolic disorder caused by genetic, epigenetic, and environmental factors [28]. Depressed and anxious individuals are at risk of developing diabetes and other clinical outcomes because of the increased risk of adopting an unhealthy lifestyle behavior, including physical inactivity, smoking, and consumption of high-fat foods [29]. Depression and chronic anxiety contribute to the impairment of a certain neuroendocrine system, the hypothalamic-pituitary-adrenal (HPA) axis, and the sympathetic nervous system; they may also cause an increase in inflammatory and platelet aggregation responses, while they are associated with insulin resistance contributing to elevated blood glucose and glycosylated hemoglobin levels (poor glycemic control) and an increased risk of metabolic complications [29].

Irrational beliefs are considered as negative and absolutistic ways of thinking about oneself, others, and society. Such beliefs may derive from early experiences and core beliefs during infancy, and they can reappear during the lifespan [6, 7]. They inevitably lead to adverse psychological outcomes (e.g., anxiety or depression) and in turn to irrational coping strategies (e.g., unhealthy nutrition, sedentary life, etc.) and eventually to negative health outcomes [7]. Based on our findings, we believe that people with a high irrational beliefs score adopt, either directly or through depression and anxiety, unhealthy dietary patterns, including high consumption of red meat and soft drinks combined with reduced intake of vegetables and olive oil. Therefore, these individuals are prone to weight gain and thereby have a high likelihood of developing type 2 diabetes and other clinical complications. Thus, we attempted to explore further psychological pathways and theories that link irrational beliefs (in addition to anxiety and emotional disorders) with diabetes and which may explain how unhealthy eating habits mediate this relationship before referring to bio-neurological mechanisms.

According to Bruch (1974) and the psychosomatic theory of eating behavior, some people interpret the sensation of sadness, emptiness, and loneliness, which are common in maladaptive thoughts and mental disorders, as similar to hunger [30]. Hence, food is used as a form of emotional comfort and an “easy strategy” to cope with distress and attain positive reinforcement [7, 30, 31]. Also, obese individuals appear to consume a higher proportion of fat [30], which may explain why participants with high irrational beliefs in our study consumed slightly more fat. The combination of irrational beliefs (and high rates of mental health problems), non-compliance with the Mediterranean diet, and attitude towards unhealthy food patterns may lead to type 2 diabetes through biochemical mechanisms. Soft drinks raise blood glucose levels, insulin resistance, and BMI, whereas high amounts of red meat contribute to insulin resistance [32, 33]. Furthermore, the fewer fruit and vegetables consumed the lower their protective role in the provision of nutrients, fibers, and antioxidants against the development of diabetes [34].

In particular, these unhealthy dietary habits and the subsequent development of obesity may be related to the deficiency of insulin secretion caused by impairment in pancreatic β-cell function when untreated, affects glucose metabolism, and results in insulin resistance, eventually leading to type 2 diabetes [35, 36]. Additionally, in line with our results, several studies have shown that type 2 diabetes frequently coexists with other metabolic abnormalities, including hypertension, hypercholesterolemia, and metabolic syndrome, which are also associated with central obesity and insulin resistance [37].

Previous studies have shown that irrational beliefs are associated with increased inflammatory processes, hypertension, and insomnia [38-40]. To the best of our knowledge, the role of irrational beliefs in predicting the risk of diabetes has been investigated by a sole cross-sectional study, showing that irrational beliefs are associated with increased diabetes risk [41].

However, our study is the first longitudinal study which systematically examined cognitive irrationality and how it affects psychological, behavioral, and clinical aspects of life according to cognitive behavioral theory. We examined the role of irrational beliefs on type 2 diabetes risk because irrational beliefs can lead to unfavorable behavioral responses such as unhealthy nutrition affecting the human metabolic profile. Since dietary habits are linked to type 2 diabetes, irrational beliefs could be strongly associated with diabetes as well.

The purpose of our study was to explore the underlying cognitive, emotional, and behavioral mechanisms that contribute to type 2 diabetes mellitus. Even though we recognize the role of genetics, neurobiology, and environment, their involvement in diabetes development is still poorly understood. There is a need for specific and modifiable factors of this costly chronic disease that has a meaningful emotional burden. By intervening and reframing irrational beliefs, individuals can better control their feelings before they become maladaptive and start practicing unhealthy lifestyle patterns to deal with their problems under an irrational perspective. Mental health literacy (i.e. knowledge and beliefs about mental disorders) is a key factor in this process. Irrational beliefs are the first stage before the development of a mental disorder and finally a physical illness. If we manage to educate people to recognize false and unhelpful thoughts during counseling or therapy, we may be able to prevent negative psychological, behavioral, and physical outcomes, including anxiety, depression, unhealthy eating habits, obesity, and type 2 diabetes.

## 5 Limitations

Person-time and incidence rates could not be calculated reliably because the exact date of diabetes development was not available in the data; the date of diagnosis was included instead. Participants with baseline cardiovascular disease (CVD), for whom information about diabetes status was not available in the data of the 10-year follow-up, were excluded from the current analysis along with 1,347 participants for whom diabetes status was not known at baseline. This may have influenced diabetes incidence.

As for diabetes determinants, relative risks were estimated by odds ratios through multiple logistic regression analysis, which may overestimate the actual relative risk. However, it has been reported that for low-prevalence diseases, odds ratio is an accurate estimate of the relative risk [42]. Another issue is that associations with disease incidence were based entirely on baseline information, but many lifestyle factors such as energy intake, physical activity, and smoking status may have changed during the long 10-year period. The study sample likely represents a limitation of the current work, although no differences were observed regarding age, sex distribution, and SES level between participants who were psychologically evaluated and the rest of the ATTICA study population. The few significant, but meaningless differences observed in baseline characteristics between those who participated in the 10-year follow-up and the rest cannot be caused by a reporting bias. Finally, we did not control for genetic causal factors apart from family history of diabetes.

## 6 Conclusions

Given that the prevalence of type 2 diabetes is rising globally, the population is aging at higher rates, and negative beliefs seem to be more frequent because of the rapid and demanding pace of life and perhaps the growth of technology, the need to promote healthier eating, especially in this group of people, is vital in diabetes prevention. Change of lifestyle behavior such as diet is not easy and may interact with several factors such as belief system and emotional state.

It will be crucial to achieve coordination among healthcare professionals by assessing irrational beliefs and providing personalized consultation to high-risk individuals including those with cognitive bias, emotional disturbance, and other clinical conditions, to intervene as early as possible in the initial stages of diabetes development.

## Abbreviations

ATP III Adult Treatment Panel III

BMI body mass index

CI confidence interval

CVD cardiovascular disease

DHA docosahexaenoic acid

FFQ food-frequency questionnaire

HPA hypothalamic-pituitary-adrenal axis

IDF International Diabetes Federation

IBI Irrational Beliefs Inventory

IPAQ Physical Activity Questionnaire

LC-PUFA polyunsaturated fatty acid

OR odds ratios

SES socioeconomic status

PSS Statistical Package for Social Sciences

STAI State-Trait Anxiety Inventory

WH waist-to-hip

WHO World Health Organization

WHt waist-to-height

ZDRS Zung Self-Rating Depression Scale
